# Clinical study design and modeling approaches to study secretion of drugs in human milk

**DOI:** 10.3389/fped.2026.1843294

**Published:** 2026-06-17

**Authors:** Prerna Dodeja, Nupur Chaphekar, Taylor Laffey, Hamdan Albukhaytan, Steve Caritis, Imam Shaik, Raman Venkataramanan

**Affiliations:** 1 Department of Pharmaceutical Sciences, University of Pittsburgh, School of Pharmacy, Pittsburgh, PA, United States; 2 Department of Obstetrics, Gynecology and Reproductive Sciences, Magee Women’s Hospital, Pittsburgh, PA, United States; 3 Department of Pharmacy & Therapeutics, School of Pharmacy, University of Pittsburgh, Pittsburgh, PA, United States; 4 Department of Pathology, School of Medicine, University of Pittsburgh, Pittsburgh, PA, United States

**Keywords:** clinical study, human milk, lactation, non-clinical models, pharmacokinetic modeling, physiologically based pharmacokinetic modeling, population pharmacokinetic modeling

## Abstract

Breastfeeding has a multitude of benefits for both the lactating mother and her infant. However, lactating women often require medications for chronic and acute medical conditions, which may pose a significant risk due to drug transfer to infants via breastfeeding. Although most drugs may pass into human milk via passive diffusion, this should rarely be a reason to completely stop breastfeeding. Drug secretion into human milk is an area of research with a paucity of data. Performing clinical studies in lactating women is essential for understanding infant drug safety. However, it is practically challenging to enroll lactating women in these studies solely for the purpose of quantifying drug transfer into human milk. In the absence of clinical studies, non-clinical models—including *in vitro*, *in vivo* (animal studies), and *in silico* (predictive modeling tools)—can provide valuable insight into drug secretion into human milk. The objectives of this review are to summarize existing pharmacokinetic metrics to quantify drug transfer to human milk and elaborate upon clinical study designs in lactation. Furthermore, we aim to explore the utility of non-clinical models to quantify drug transfer into human milk, given the absence of clinical data. Finally, we discuss *in silico* models, including empirical equations used to derive milk-to-plasma ratios, as well as pharmacokinetic models to predict and analyze drug concentration-time profiles during lactation.

## Introduction

Lactation is the process of producing and releasing milk from the mammary glands located in human breasts. The process of lactation is triggered during pregnancy when hormonal changes signal the mammary glands to produce milk (1). Breastfeeding, or nursing, is the process by which milk is fed to an infant. There are a multitude of benefits of breastfeeding instead of utilizing formula. Given that human breast milk includes immunoglobulins, such as Immunoglobulin A (IgA), which defend against respiratory infections (2), it is believed to offer the most comprehensive array of bioactive factors for development of immune response (3). Medication use during lactation is common, with an estimated 66%–72% of nursing mothers on some form of medication (4). Some amount of the mother’s medication may pass into human milk, potentially exposing the infant to xenobiotics unnecessarily. Evidence-based information regarding the use of medication in lactation is lacking. In 2023, a retrospective analysis of 422 New Molecular Entities (NMEs) found that only 2% of medications had a key advisory statement supporting their use during pregnancy or lactation. Pregnant and lactating individuals are advised against the use of drugs in nearly one-half of newly approved medications. Data for 194 NMEs (46%) for drugs in lactation were classified as “against use” including emtricitabine, and rosuvastatin calcium; with only 3 medications (1%) with evidence “supportive of use” during breastfeeding, including perflutren and plecanatide (5). Factors driving the secretion of drugs into human milk have been discussed in Part 1 of the current review series (1). A list of abbreviations within this review has been presented in Table 1.

**Table 1:** Table of abbreviations.

Abbreviation	Definition
IgA	Immunoglobulin A
NME	New Molecular Entities
US FDA	United States Food and Drug Administration
PLLR	Pregnancy and Lactation Labeling Rule
PMR	Post-Marketing Requirement
M/P	Milk to Plasma Ratio
RID	Relative Infant Dose
IDD	Infant Daily Dose
MeSH	Medical Subject Headings
WHO	World Health Organization
${\text{AUC}}_{\mathrm{milk}}$	Area Under the milk concentration-time Curve
PK	Pharmacokinetics
UAR	Upper Area under the curve Ratio
PBPK	Physiologically Based Pharmacokinetic Modeling
AUC	Area Under the concentration-time Curve
MCF10F	Michigan Cancer Foundation Epithelial Cell Line
CIT3	Third Citrate Synthase Gene
MEC	Mammary Epithelial Cell
CYP450/ CYP	Cytochrome P-450
CS	Caesarean Section
$C_{\max}$	Maximum Concentration of drug in plasma
$C_{ss}\text{Average}$	Average Concentration of drug in plasma at steady state
$C_{\mathrm{trough}}$	Concentration reached by a drug immediately before the next dose is administered
$T_{\max}$	Time to reach $C_{\max}$
QSAR	Quantitative Structure Activity Relationship
MLP	Multilayer Perceptron
QSPR	Quantitative Structure Property Relationship
SVM	Support Vector Machine
C-C	Carbon-Carbon
E-E	Electron-Electron
$CL_{\sec}$	Secretion Clearance
$CL_{\mathrm{re}}$	Reuptake Clearance
BCRP	Breast Cancer Resistance Protein
PFOA	Perfluorooctanoic Acid
PFOS	Perfluorooctane Sulfonic acid
TCE	Tetrachloroethylene
EPA	Environmental Protection Agency
PopPK	Population Pharmacokinetics
CYP2D6	Cytochrome P-450 2D6
AAG	Alpha-1-Acid Glycoprotein
DolPHIN-1	Dolutegravir in Pregnant mothers with HIV Infection and their Neonates
HIV	Human Immunodeficiency Virus
mRNA	messanger RiboNucleic Acid
$C_{\mathrm{avg}}$	Average Concentration of drug in plasma
${\text{AUC}}_{0-24}$	Area Under the Curve over 24-h dosing
GI	GastroIntestinal tract
CMT	Compartment

It is safe to assume that most drugs will transfer to human milk via passive diffusion (6). However, maternal drug therapy should rarely be a reason to stop breastfeeding (7, 8). This is a Catch-22 situation, since clinicians may not be aware which drugs are potentially harmful to the infant and which are safe (9). Based on a recent meta-analysis of pregnancy and lactation labels, the sources of lactation data in drug labels are studies in animals (59%), other studies (8%) followed by studies in humans (5%) (5, 10). Therefore, pregnant and lactating women are cautioned against the use of nearly half of newly approved medications primarily based on evidence from animal studies. The limited human data found in the literature is sourced from case studies, contributing to the information desert. The United States Food & Drug Administration (US FDA) enacted the Pregnancy and Lactation Labeling Rule (PLLR) in 2015, establishing an updated criteria for incorporation of data on use of prescription medication and biological products in pregnant and lactating women. This act stimulated inclusion of this population in clinical studies, as evidenced by a recent meta-analysis demonstrating increasing trends in Post Marketing Requirement (PMR) filings for pregnancy and lactation studies post 2015 (11). Although conducting clinical studies in lactating women is the best approach to fill knowledge gaps, it comes with many practical challenges. Enrollment of pregnant and lactating women has numerous logistical problems (12). Moreover, there are ethical concerns regarding the risk-benefit ratio of certain medications for the mother and infant. Importantly, rather than excluding lactating women from clinical studies, their inclusion is essential for model validation and generation of real-world pharmacokinetic data. There is currently a lack of standardized guidelines for conducting clinical lactation studies, and the development of more robust frameworks is urgently needed. Recent regulatory efforts by the FDA and EMA have emphasized the importance of including pregnant and lactating individuals in drug development programs. The UmbrelLACT study protocol, recently proposed by Van Neste et al., represents a significant step toward standardizing lactation pharmacokinetic study design and enabling cross-study harmonization of data (13).

Clinical data is necessary to make appropriate conclusions about drug transfer to milk. Primary pharmacokinetic parameters of interest derived from clinical studies include the milk-to-plasma ratio (M/P) and the relative infant dose (RID). The ratio of drug levels in the human milk to the drug concentrations in plasma, known as the M/P is the primary metric to determine relative partitioning of drugs from plasma to human milk. The advantage of this metric is that while most drugs pass into human milk via passive diffusion, they may not accumulate in the human milk, which is the primary safety concern. Further, in order to assess concerns related to infant safety, metrics such as infant daily dose (IDD) and RID are used. The first, IDD, is based on the average drug levels in human milk and daily volume of milk ingested by a breastfed infant (Equation 1). The second, RID, compares the infant drug exposure received via human milk relative to the dose taken by the mother, and is expressed as a percentage (Equation 2).

Given the absence of meaningful clinical data, modeling has greatly contributed to a deeper understanding of the passage of drugs and xenobiotics into human milk (14). There are numerous reviews in the literature that provide specific examples of drug transfer to human milk and general modeling approaches (15–17). However, our focus is on providing an updated overview of clinical and non-clinical approaches to predict the milk-to-plasma ratio, how *in vitro* or predictive methods compare to observed human data, and the current status of pharmacokinetic models built for lactation. Part 1 of the literature review was focused on the practical considerations in sample collection, storage and bio-analytical quantification of drugs in human milk (1).

The objectives of the current review are : (1) summarize and evaluate existing pharmacokinetic metrics to quantify drug transfer into human milk; (2) explore the utility of non-clinical models to quantify drug transfer into milk; (3) elaborate upon existing clinical study designs and their limitations; and (4) comprehensively summarize and evaluate *in silico* models, including empirical equations used to derive milk-to-plasma ratio, and pharmacokinetic models to predict drug concentration-time profiles in human milk.

### Literature search methods

This manuscript is presented as a structured narrative review informed by a systematic literature search. MeSH keywords, alone or in combination: “lactation”, “human milk”, “clinical studies”, “pharmacometrics”, “milk-to-plasma ratio”, “modeling” were used. Three independent reviewers (P.D, N.C and H.A) extracted clinical, pharmacometric, and non-clinical studies by querying PubMed and Web of Science, followed by evaluation of relevant bibliographies and organizational websites. Eligibility criteria encompassed peer-reviewed articles reporting pharmacokinetic data, non-clinical models, or predictive approaches relevant to drug transfer into human milk; studies were screened by title and abstract, followed by full-text review. Relevant literature published until December 2024 was included; the search was originally conducted through October 2023, and select 2024 publications identified during manuscript preparation were subsequently incorporated.

## Drug exposure metrics

**Figure 1:**
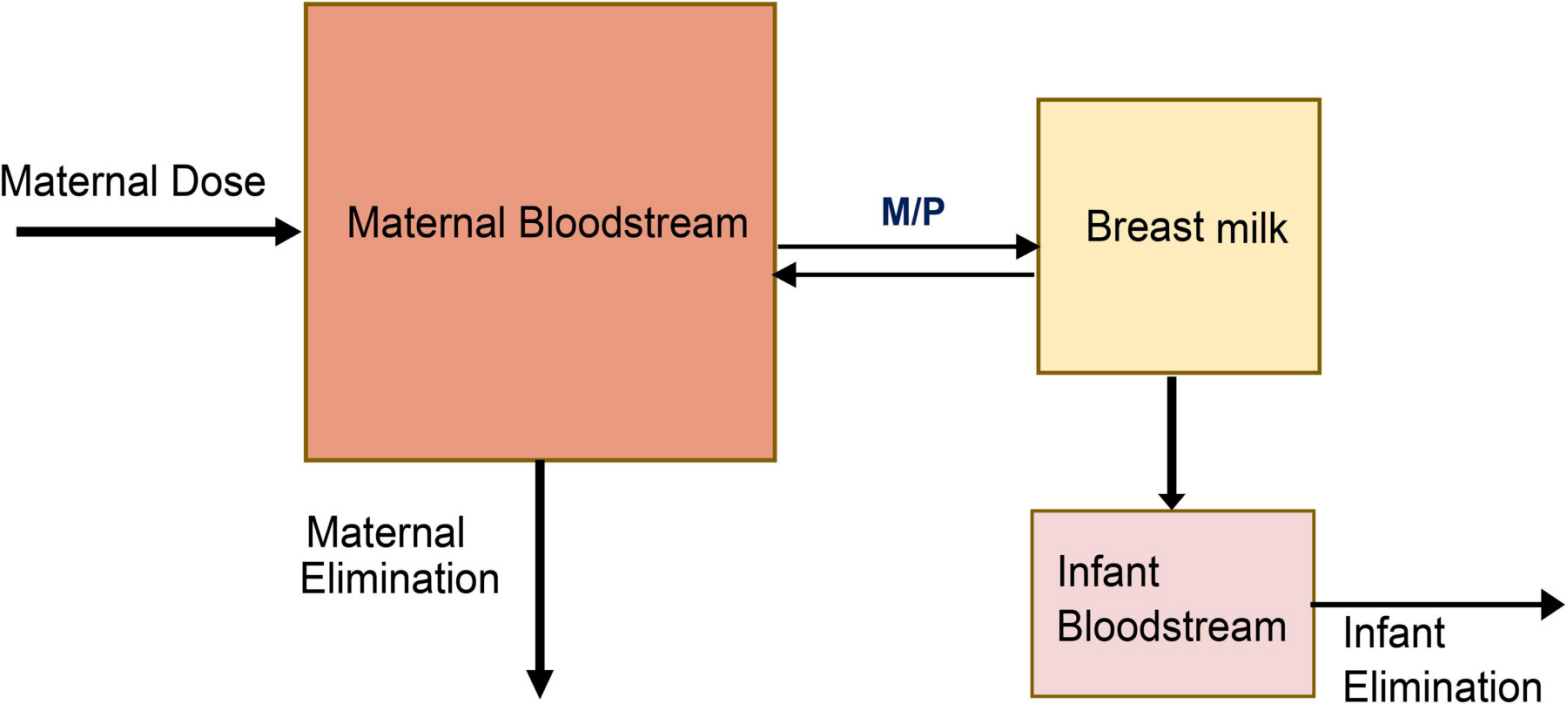
Schematic representation of a two-compartment pharmacokinetic model describing drug transfer from the maternal bloodstream to breast milk and subsequent infant exposure [Figure 1 has been adapted from (16)].

In a lactation clinical study, the drug will be ingested by the nursing mother, and human milk will be collected at several specified time points to estimate the drug concentrations. This data will subsequently be used to calculate the ${\text{AUC}}_{\mathrm{milk}}$. Optimal sample collection and storage have been discussed previously in Part 1 of the review (1). Figure 1 depicts a schematic two compartment pharmacokinetic (PK) model to assess the passage of the drug to the infant. Once the drug concentration in human milk is known, the Infant Daily Dose (IDD) can be calculated using the following equation:

$$\text{Infant Daily Dosage}\;(\text{mg/kg/day})=\frac{C_{\mathrm{milk}}\;(\text{mg/L})\cdot V_{\mathrm{milk}}(\text{L})}{\text{Infant Weight}\;(\text{kg})}$$ (1)

where $C_{\mathrm{milk}}$ refers to the drug concentration in milk and $V_{\mathrm{milk}}$ refers to the daily (24-h) volume of milk ingested by the suckling infant. For simplicity, most IDD calculations estimate the $V_{\mathrm{milk}}$ by dividing the weight-adjusted daily milk intake of 150 mL/kg/day by the feeding frequency (18). Based on this value, we can further calculate the RID, proposed by the World Health Organization (WHO) working Group (19, 20):

$$\text{Relative Infant Dose}\;(\%)=\frac{\text{Infant Daily Dosage}\;(\text{mg/kg/day})}{\text{Maternal Daily Dosage}\;(\text{mg/kg/day})}\cdot 100$$ (2)

Beyond RID, the estimated IDD should be contextualized against the approved pediatric dosage of the drug, when available. When a drug lacks approved pediatric labeling—as is the case for many medications taken by lactating women—the RID threshold remains the primary safety benchmark, underscoring its continued clinical utility despite its inherent limitations. The WHO working group proposed that drugs with an RID >10% of the lower end of weight-adjusted relative dose may not be safe, and those with an RID >25% should be completely avoided in lactating women. Drugs with an RID <10% are generally considered to be safe during lactation, while keeping the nature and safety profile of the drug in mind. However, there are several pitfalls with the use of RID since it is an arbitrary cutoff value. RID is influenced by a multitude of parameters, including milk intake, feeding intervals, intervals between maternal dosing and feeding, and drug concentrations in the milk, all of which can contribute to variability in the estimate (21). RID must be interpreted with caution, taking into account the pharmacological characteristics of the drug and the potential for adverse effects on the infant. RID is not itself a toxicity metric since it does not account for the infant’s oral bioavailability, age and developmental stage, degree of prematurity, hepatic and renal maturation, or the contribution of active metabolites which substantially influence the relationship between ingested dose and actual systemic exposure in the infant. These concerns have been discussed in detail previously by Anderson & Sauberan (16). A more mechanistically informative approach to infant risk assessment is the Relative Infant Exposure (RIE), defined as the ratio of infant AUC to maternal AUC. This AUC-based metric captures the integrated pharmacokinetic relationship between mother and infant and is inherently less susceptible to the time-dependent variability that limits point-estimate metrics such as RID. Recently, Yeung et al. described a novel risk assessment metric called the Upper Area Under the curve Ratio (UAR) (22). The UAR is defined by dividing the 95th percentile of simulated pediatric area under the curve (AUC) by the median adult therapeutic AUC. These simulated pediatric AUCs are obtained using a physiologically based-pharmacokinetic model (PBPK) is discussed in this section. The UAR metric is an alternative to relative infant exposure assessment, and it’s applicability has been demonstrated using a PBPK model for lamotrigine (23).

### Quantification of milk-to-plasma ratio

The accuracy of the RID estimate depends on the accuracy of the estimation of each of the parameters in Equation 1 and M/P. As discussed earlier, we can address this via direct measurement of drug concentrations in human milk. Subsequently, the IDD can be extrapolated to other patients using the M/P (17).

However, there are two major concerns. Firstly, enrollment of women postpartum in a lactation study is practically challenging. This is compounded by the fact that they will be donating precious human milk, reducing the volume given to their child. Secondly, the accurate measurement of M/P is influenced by extrinsic factors. M/P is not a constant for any given drug that can be reliably calculated, it varies significantly depending on several factors. These include: the time of clinical sampling after maternal drug administration, whether the drug is given as single or multiple doses, route of drug administration, composition of milk, and whether foremilk or hindmilk was analyzed (24). The calculation of M/P is the key starting point for the development of more sophisticated models. The single largest pitfall in the calculation of M/P is the assumption that drug concentrations in human milk and plasma will parallel each other throughout the dosing interval (one constant M/P). However, in reality, drug concentrations in maternal plasma and human milk do not fall and rise in parallel, and different ratios are obtained at different time points. Wilson et al. have comprehensively covered this phenomenon by simulating M/P to demonstrate model- and time-based effects on the M/P. We refer the reader to articles detailing the pitfalls in estimating M/P (16, 25, 26). Although the use of single-point estimates of M/P ratios has been discredited, most clinical studies report point estimates of M/P from opportunistic human milk and plasma samples obtained from patients. From a clinical standpoint, it is important to note that the M/P alone cannot be used to predict the safety of a drug while breastfeeding. The most useful M/P obtained would be from a full concentration-time profile of both maternal plasma and milk to derive the AUC for each, therefore estimating an $M/P_{\mathrm{AUC}}$ (14). Collection of human milk from lactating mothers is important not just for pharmacokinetic studies, but also as a reference point for predictive modeling approaches. These models can be validated against measurements in human milk; therefore, it is critical to collect and analyze human milk samples accurately (15).

However, enrolling lactating women is difficult unless they are already part of a long term longitudinal pregnancy study, such as those conducted for antiretroviral agents (27, 28). Moreover, for the purpose of determining the M/P, and understanding the pharmacokinetics of a drug in human milk, justifying the conduct of such studies can prove to be challenging. Therefore, as a starting point, leveraging non-clinical models (*in vitro*, *in vivo*) can provide invaluable information regarding drug characteristics and inform potential for drugs partitioning to milk. Inadequacies in extrapolation of *in vitro* or animal */in vivo* data to predict clinical scenarios must be kept in mind. A pragmatic combination of non-clinical models with PK modeling tools is an innovative strategy to make reliable predictions of drug PK in human milk.

## Non-clinical models

In the absence of dedicated clinical lactation studies, non-clinical experimental approaches represent an important starting point for evaluating the potential for drug transfer into human milk. These approaches include *in vitro* systems and *in vivo* animal models, which are primarily used to characterize passive diffusion, permeability across mammary epithelium, and the influence of physicochemical drug properties on milk partitioning (29). While non-clinical models cannot replicate the dynamic physiology of human lactation, they are valuable for qualitative risk ranking, mechanistic hypothesis generation, and parameterization of pharmacokinetic modeling frameworks rather than for direct quantitative prediction of infant exposure.

Among *in vitro* approaches, equilibrium dialysis techniques and mammary epithelial cell-based permeability assays are most commonly employed. The Visking tube dialysis method estimates milk-to-plasma ratios by allowing diffusion of drug across a semi-permeable membrane separating milk and plasma matrices (30). Using this approach, Notarianni et al. evaluated caffeine, cimetidine, diazepam, indomethacin, estradiol, phenytoin, and warfarin. As summarized in Table 2, M/P ratios for five of the seven compounds fell within clinically reported ranges, whereas diazepam and warfarin exceeded observed human values. These findings demonstrate the utility of dialysis-based assays as screening tools while highlighting their limitations in capturing milk-specific binding and partitioning processes.

**Table 2:** Milk-to-plasma (M/P) ratios estimated using equilibrium dialysis (Visking tube) compared with clinically observed values.

Drug	M/P (*in vitro*)	Observed Clinical M/P
Caffeine	0.79	0.44–11.8
Cimetidine	0.86	0.44–11.8
Phenytoin	0.11	0.13–0.45
Indomethacin	0.007	≤1.48
Estradiol	0.11	0.07–0.30
Diazepam	**0.50**	0.11–0.27
Warfarin	**0.58**	<0.0124

Bolded values indicate M/P estimates falling outside reported clinical ranges.

Cell-based models introduce greater biological relevance by incorporating mammary epithelial barriers. Zhang et al. utilized human mammary epithelial cells cultured in Michigan Cancer Foundation Epithelial Cell Line (MCF10F) medium to assess drug permeability and predict M/P ratios (31). Predicted values showed good agreement with clinical observations for loratadine, while variable concordance was observed for other compounds (Table 3), underscoring the compound-dependent performance of these systems and the need for broader validation across diverse drug classes.

**Table 3:** Comparison of predicted milk-to-plasma (M/P) ratios obtained using mammary epithelial cell (MEC) models with clinically observed values.

Drug	Model system	Predicted M/P	Observed clinical M/P
Clarithromycin	MCF10F	**0.33**	0.81
Digoxin	MCF10F	**4.72**	2.9
Doxorubicin	MCF10F	**1.01**	2.32
Loratadine	MCF10F	0.87	0.87
Pefloxacin	MCF10F	**2.36**	1.11
Venlafaxine	MCF10F	**1.84**	4.59
Rifampin	CIT3 MEC	0.20	0.23
Theophylline	CIT3 MEC	0.70	0.61
Paracetamol	CIT3 MEC	1.00	0.87
Aspirin	CIT3 MEC	0.03–0.08	0.03

Bolded predicted values indicate notable deviation from observed clinical M/P.

Similarly, Athavale et al. employed CIT3 mouse mammary epithelial cells (MECs) to estimate M/P ratios for rifampin, theophylline, paracetamol, and aspirin (32). Comparison with clinically reported values demonstrated close agreement (Table 3), supporting the use of MEC-based assays as qualitative tools for assessing milk transfer potential and informing model assumptions.

Beyond *in vitro* systems, animal models have been used to evaluate drug metabolism and milk transfer (33). However, species-specific physiological differences limit direct extrapolation of animal-derived M/P ratios to humans (34). As shown in Table 4, mouse-derived M/P ratios frequently overestimate milk transfer relative to clinical observations, particularly for basic and lipophilic compounds such as cimetidine, acyclovir, and quetiapine. In contrast, closer agreement is observed for a limited number of drugs (e.g., metoprolol and trazodone), indicating that concordance is compound-dependent rather than systematic. These discrepancies reflect predictable inter-species differences in milk composition, pH, lipid content, and systemic pharmacokinetics (35). A summary of the non-clinical models to predict M/P ratio is presented in Table 5.

**Table 4:** Comparison of milk-to-plasma (M/P) ratios observed in mouse models and humans, illustrating inter-species variability.

Drug	Observed M/P (Mouse)	Observed M/P (Human)
Cimetidine	**10.33**	4.18
Atenolol	**1.99**	3.12
Disopyramide	**1.78**	2.82
Metoprolol	2.98	2.79
Acyclovir	**4.21**	1.58
Cefotaxime	**0.22**	0.08
Propylthiouracil	**5.84**	0.13
Trazodone	0.20	0.14
Praziquantel	**1.42**	0.24
Quetiapine	**1.11**	0.36

Bolded mouse M/P values indicate substantial divergence from human observations.

**Table 5:** Overview of non-clinical approaches to quantify M/P.

Name of drugs	Type of experiment	Model	Procedure	Comparison with human data	Refs.
Caffeine, Cimetidine, Diazepam, Indomethacin, Phenytoin, Warfarin	*In vitro*	Visking Tube	Visking Tube: permeable membrane which allows the solution components to diffuse	Diazepam and Warfarin M/Ps overpredicted (Table 2)	(30)
P-glycoprotein substrates: Clarithromycin, Digoxin, Doxorubicin, Loratadine, Pefloxacin, Venlafaxine	*In vitro*	Human MEC (HMEC)	HMEC treated with MCF101 medium	Doxorubicin, Clarithromycin and Pefloxacin under-predicted (Table 3)	(31)
			Measure permeability of drugs through monolayer	Digoxin over-predicted	
27 Drugs	*In vivo*	Cassette Dosing- Mice	Micro-osmotic pumps implanted peritoneally into lactating mice	Species-specific differences in milk protein and lipid content limit concordance (Table 4)	(35)
			Milk collected from nursing mice 60-hr after implantation		
Probe Drugs	*In vitro*	Liver slices - Rat and Mouse	Determine CYP messanger Ribonucleic Acid (mRNA) Levels using reverse transcription-polymerase chain reaction	Species-specific isoforms of CYP1A1, CYP1A2, CYP2B1 and CYP3A1 show appreciable interspecies differences in terms of catalytic activity.	(33)
			*in vitro* measurements compared to induction data from *in vivo* experiments		
Olanzapine	*In vivo*	PK Study in Sheep	Six nursing sheep and their lambs treated with drug for 10 days.	Sheep have lower plasma drug concentrations.	(36)
			Blood and milk samples collected, additional samples taken at steady state.		

### Limitations of non-clinical models

Despite their utility, non-clinical models have important limitations. A fundamental challenge in translating non-clinical data to humans is the lack of reliable data on mammary epithelial surface area, which is a critical parameter for estimating transepithelial drug clearance. Discrepancies in surface area estimates have been noted between studies; for example, the Job et al. (2020) (96) analysis of ondansetron and the Zhang et al. (2020) (97) PBPK model for BCRP substrates used substantially different assumptions regarding mammary surface area, contributing to divergent clearance predictions. Standardization of this parameter will be important for improving the quantitative reliability of both *in vitro* and mechanistic PBPK models of lactation. Most *in vitro* systems are also designed to characterize passive diffusion and are unable to adequately quantify active transport processes across mammary epithelium. While passive diffusion dominates milk transfer for many drugs, transporter-mediated secretion may be clinically relevant for select compounds. Failure to account for such mechanisms may contribute to systematic under- or over-prediction of milk concentrations in both experimental and animal models.

Rodent models are commonly used to estimate milk transfer; however, their translational relevance is constrained by substantial physiological differences. Milk pH differs between species, with reported values of approximately 7.4 in human milk and 6.5 in mouse milk (35), influencing drug ionization and ion trapping depending on drug pKa. In addition, milk lipid content is markedly higher in rodents (mouse: ∼11.4%; rat: ∼15%) than in humans (∼4%), which may disproportionately inflate M/P ratios for lipophilic compounds. Species-specific differences in drug metabolism, particularly cytochrome P450 enzyme expression, further complicate extrapolation by altering systemic exposure and indirectly affecting milk concentrations (33). Collectively, these factors limit the reliability of rodent models for quantitative prediction of human milk exposure (35, 37).

Given these limitations, alternative species with milk composition and physiology more comparable to humans have been explored. Ősz et al. evaluated olanzapine transfer into milk in lactating sheep and reported M/P ratios ranging from 0.1 to 0.9 (mean 0.46), comparable to observed human data and values predicted using empirical equations (36, 38). These findings suggest that sheep models may serve as useful proof-of-concept systems for select compounds, particularly when integrated with empirical or PBPK modeling approaches.

Overall, non-clinical models provide valuable qualitative insight into drug partitioning into human milk but are insufficient as standalone predictors of infant exposure. Their greatest utility lies in bounding parameter uncertainty, and supporting PBPK and population pharmacokinetic frameworks that integrate experimental data with human physiology to enable quantitative risk assessment.

## Clinical studies and design considerations

Human lactation studies investigating drug pharmacokinetics and pharmacodynamics must be carefully designed with several factors in mind, including the research question and study objectives. Additional key considerations include the drug dose, route of administration, and dosing frequency. Studies involving drugs used for acute medical conditions or drugs that do not accumulate in the body despite chronic use, may be designed as single-dose studies. However, for chronic drug use requiring multiple dosing, the FDA recommends that subjects be studied at steady state (the time when the concentration of the drug in the body remains constant) (39). The FDA Clinical Lactation Study Guidance classifies human lactation studies as follows (40):

### Lactating women design

When only human milk samples, or human milk and maternal plasma samples are sufficient to address the research question, then the “lactating women only” design may be appropriate. Depending on the availability, the study team may collect only human milk or simultaneous maternal plasma and human milk samples. These studies exclude infant plasma sampling, therefore they cannot be used to quantify systemic drug exposure in the infant.

### Milk only design

Only lactating women are enrolled in studies in this design, and the only sample collected is human milk. Human milk may be collected at a single predetermined time point or multiple times over a specified dosing interval. For multiple sampling, small volumes of milk may be collected frequently throughout the dosing interval, (e.g., over 24-h), Milk-only studies are useful for detecting the presence of a drug and its metabolites in human milk, the effect of a drug on milk production and composition, and determining the time interval between maternal drug administration and drug passage into human milk. For most drugs taken during pregnancy and lactation, a milk-only study is sufficient (15).

### Plasma and milk design

A plasma and milk study design is useful to determine the drug concentration-time profile and basic PK parameters of the drug administered to the mother. This study can also facilitate dose optimization during the postpartum time period. Nitsun et al. collected human milk and maternal plasma samples from five mothers to determine the pharmacokinetics of midazolam, propofol, and fentanyl (41). The basis for this study came from the recommendation for lactating women undergoing surgery under general anesthesia who were required to pump and discard their milk for 24 h after the procedure. In 24 h of milk collection, they found an average of 0.005% of the maternal midazolam dose, 0.027% of the propofol dose, and 0.033% of the fentanyl dose were collected in human milk (41). Similarly, the degree of benznidazole transfer into human milk in lactating women was investigated by Garcia-Bournissen et al. (42). Samples were collected before the first dose and on the 7th and 30th day after starting benznidazole treatment. Milk and plasma concentrations, M/P , and median RID were reported (42). The maternal milk and plasma sampling allow the M/P to be estimated, which will be useful for understanding the extent of transfer from plasma to human milk for a drug and can be used to predict milk exposure at various dose levels of the drug. M/P and RID cannot be determined from a milk-only design, since RID has to be calculated using estimations of infant milk intake (Equation 2), which could lead to over- or under-estimation of this parameter.

### Mother-infant pair design

These studies involve collection of maternal samples (plasma, human milk) and corresponding infant samples (plasma and/or urine) over a specified dosing interval. Data from these studies can determine the concentration-time profile and pharmacokinetics of a drug in lactating women, and the amount of drug excreted into human milk. In addition, this type of study can estimate potential absorption of drugs in breastfed infants, leading to an understanding of systemic exposure in the infant. This design should be considered only if prior information is available about the extent of drug transfer into human milk. The investigator may further explore how the drug may affect the infant exposed to it. Seaton et al. studied the relationship between maternal ingestion of oxycodone after cesarean section (CS) and the resultant maternal plasma, human milk, and neonatal plasma drug levels up to 72-h postpartum (43). Fifty lactating mothers had plasma and human milk samples analyzed at 24-h intervals after CS and fourty-one neonates had plasma samples taken 48-h post CS. A significant correlation between maternal plasma and milk drug levels and the M/P was reported. The investigators noted the differences in relationship between plasma and milk levels over 48-h. Oxycodone was detected in the one infant’s plasma. The authors concluded that oxycodone can be detected in human milk up to 72-h postpartum and that breastfed infants may receive > 10% of the therapeutic dose, but that lactation poses minimal risk since small volumes of human milk are ingested during this period (43).

### Longitudinal design

For chronic drug use, this study design is appropriate. It involves comparing samples collected from the lactating mother and/or the infant at different postpartum time periods. For example, plasma or human milk samples collected from a lactating woman post delivery, and at the initiation of breastfeeding may be compared with samples from the same woman collected later in the postpartum period. Each lactating woman serves as her own control in the longitudinal design. Longitudinal studies have most commonly been conducted for pregnant women living with HIV who are on antiretroviral therapy (44).

### Multiple arm design

Samples collected from multiple lactating women at different postpartum periods are compared with samples from matched healthy lactating women as control. This design is commonly used for drugs that are administered acutely over a short period of time.

## Ideal study design

**Figure 2:**
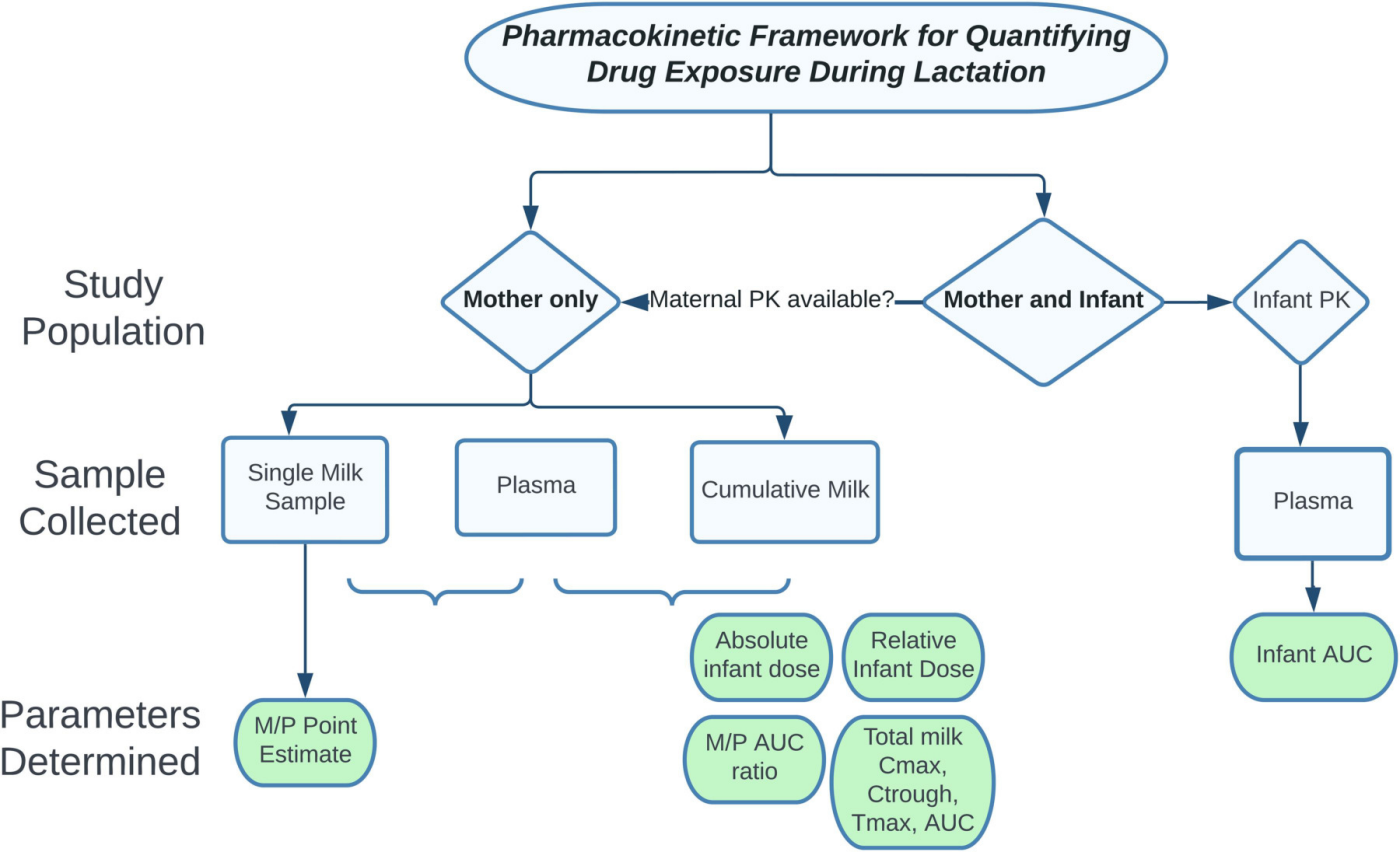
Pharmacokinetic framework for quantifying drug exposure during lactation, illustrating study design pathways based on study population (mother only vs. mother–infant) and available biological samples. For maternal-only studies, collection of single milk samples enables estimation of milk-to-plasma (M/P) point ratios, while paired plasma and cumulative milk sampling allow estimation of integrated exposure metrics including M/P AUC ratio, absolute infant dose, and relative infant dose, as well as milk pharmacokinetic parameters (e.g., $C_{\max}$, $C_{\mathrm{trough}}$, $T_{\max}$, AUC). In mother–infant studies, infant plasma sampling enables direct characterization of infant exposure (e.g., infant AUC) (25, 40, 46). $C_{\max}$, maximum concentration; $C_{\mathrm{trough}}$, concentration immediately prior to the next dose; $T_{\max}$, time to reach $C_{\max}$; AUC, area under the concentration–time curve; M/P, milk-to-plasma ratio.

In the absence of logistical and practical concerns, the ideal study design would be a longitudinal, mother-infant pair approach. By comparing drug concentrations in the same mothers and neonates over time, conclusions about the cumulative absorption of the drug in infants can be made. It also allows for each pair to serve as their own control group over time; hence, no major assumptions need to be made while comparing to a different lactating woman control group. In an ideal world, this approach would be utilized, but this study design may not always be feasible. Begg et al. outlined a decision tree schema for studies to measure the amount of drug in human milk (25). We further built upon this schema to provide a simplified decision-tree for choosing the most appropriate study based on available samples and parameters to be estimated to answer the research question, presented in Figure 2. Additionally, the Milk4Baby decision tree—a pragmatic framework developed as part of the ConcePTION project—offers contextualized method selection for assessing infant systemic exposure and is particularly suited to real-world clinical scenarios where resources and sampling opportunities are constrained (45).

## Predictive models for M/P

Models can be used to characterize how lactation alters maternal pharmacokinetics and to quantify drug transfer into human milk. From a compartmental modeling perspective, lactation may influence maternal volume of distribution ($V_{d}$) and clearance (CL), particularly when human milk is represented as an additional peripheral pharmacokinetic compartment (Figure 1). In parallel, empirical modeling approaches leverage physicochemical drug properties to predict milk-to-plasma ratios, providing an alternative means to estimate drug passage into human milk when direct clinical data are unavailable. Due to the complex nature of human milk, several physicochemical factors have to be taken into account while predicting M/Ps. Rasmussen et al. were the first to emphasize the importance of pH differential between milk and plasma based on studies in goats and cows (47–49). There are four major models to predict drug concentrations in human milk, as follows:

1. Unbound Drug Distribution Model
  The unbound drug distribution model is based off the Henderson-Hasselbach equation to derive the Mu/Pu ratio which refers to the measured milk:plasma unbound drug ratio (37)
  $$\text{Acidic Drug Mu/Pu}=\frac{1+10^{\left({\text{\textbackslash{},pH}}_{m}-{\text{\textbackslash{},pK}}_{a}\right)}}{1+10^{\left({\text{\textbackslash{},pK}}_{a}-{\text{\textbackslash{},pH}}_{\;p}\right)}}$$ (3)
  $$\text{Basic Drug Mu/Pu}=\frac{1+10^{\left({\text{\textbackslash{},pK}}_{a}-{\text{\textbackslash{},pH}}_{m}\right)}}{1+10^{\left({\text{\textbackslash{},pK}}_{a}-{\text{\textbackslash{},pH}}_{\;p}\right)}}$$ (4)
  where ${\text{\textbackslash{},pK}}_{a}$ is the negative base-10 logarithm of the acid dissociation constant($K_{a}$), ${\text{\textbackslash{},pH}}_{m}$ and ${\text{\textbackslash{},pH}}_{\;p}$ are the pH values of milk and plasma, respectively. Equations 3 and 4 adequately predict the ratio of unbound drug in human milk to that in plasma, but cannot be used to predict M/P adequately. The unbound distribution model is too simplistic to be utilized practically.
2. Membrane Diffusional Model
  To improve upon the previous model, Meskin and Lein (50) also considered two factors affecting the ability of a molecule to pass through membranes: molecular weight ($M_{w}$) and octanol-water partition coefficient (LogP). They utilized step-wise regression analysis to test M/Ps and physicochemical parameters of 20 acidic and 15 basic drugs to derive the following relationships:
  Acidic Drugs:
  $$\text{Log}\;M/P=2.068-0.162\sqrt{M_{W}}-0.185\log P$$ (5)
  Basic Drugs:
  $$\text{Log}\;M/P=0.265-0.153\log P-0.128\;\text{Log}\frac{U}{D}$$ (6)
  where U/D is the ratio of undissociated or unionized to dissociated or ionized drug. Equations 5 and 6 also tend to fall short, since they do not take into account any factors related to human milk and only account for membrane transfer.
3. Phase Distribution Models
  (a) Fleishaker Model
    The previously described models take into account membrane characteristics and drug pH and pKa. However, the missing piece of the puzzle are the components of human milk itself. Fleishaker et al. incorporated the binding of drugs to proteins in milk and plasma, as well as the creamatocrit ratio ($C_{t}$) which refers to the fractional component of fat in a given volume of milk (51). The phase distribution model enables the calculation of M/P given that the Skim milk to Whole milk concentration ratio (S/M) is known.
    $$M/P=\frac{\;f_{\;p}\cdot f_{\;p}^{un}}{f_{m}\cdot f_{m}^{un}(S/M)}$$ (7)
    where
    $$S/M=\frac{1}{1+C_{t}(f_{m}K_{\;f}-1)}$$
    and $f_{\;p}$ and $f_{m}$ are the fractions unbound in plasma and milk, $f_{\;p}^{un}$ and $f_{m}^{un}$ are the fractions unionized in plasma and milk and $K_{\;f}$ is the milk fat to water partition coefficient.
    This model demonstrates improved accuracy compared to prior models described; however, the parameters in Equation 7 require *in vitro* experiments to be conducted to measure milk protein binding and fat partitioning. This may be a major deterrent to adoption of model-based approaches: conduct of *in vitro* experiments to derive an estimate.
  (b) Atkinson & Begg Model
    Atkinson & Begg developed a similar model; however, instead of using S/M which is not known for most drugs, they utilized the plasma protein binding values. Based on known plasma protein binding of drugs, an equation was developed to estimate the milk protein binding value (52). The resultant phase distribution model presented into the following equation:
    $$M/P_{\mathrm{phase}}=F_{u,p}\cdot \frac{\text{Mu}}{\text{Pu}}\cdot \left(\frac{0.955}{F_{u,m}}\right)+0.045\text{milk lipid}P_{\mathrm{app}}$$ (8)
  or
    $$M/P_{\mathrm{phase}}=F_{u,p}\cdot \frac{\text{Mu}}{\text{Pu}}\cdot K$$ (9)
    where
    $$K=\left(\frac{0.955}{F_{um}}\right)+0.045\text{milk lipid}P_{\mathrm{app}}$$
    and $F_{u,p}$ and $F_{u,m}$, in Equation 8 are the unbound fractions of the drug in plasma and milk, respectively, and the milk lipid $P_{\mathrm{app}}$ is $C_{\mathrm{lipid}}/C_{\mathrm{ultrafiltrate}}$ which refers to the milk lipid: ultra filtrate partition coefficient at pH 7.2. $\frac{M_{u}}{P_{u}}$ in Equation 9 refers to the ratio of unbound concentrations of drug in milk and plasma, as predicted by the Henderson Hasselbach equation. The values of 0.955 and 0.045 refer to the mean value of the composition ratio of drug in the aqueous and lipid phase, respectively. Conceptually, the Fleishaker and Atkinson & Begg models are essentially identical (Equations 7 and 8). The phase distribution model overestimates the M/P for acidic drugs and underestimates M/P for basic drugs (17).
  (c) Log-transformed Phase Distribution Model
    Step-wise multiple regression analysis was adopted to the phase distribution model mentioned above so that the weight of each component could be assessed (14). The natural logarithm transformed formula of Equation 8 is the log-transformed phase distribution model. It was found to exhibit the best predictive performance so far (53, 54). We must keep in mind that the log-transformed phase distribution model is a regression model, while the previous Fleishaker et al. and Atkinson & Begg models were derived to utilize *in vitro* information for the prediction of M/P (17). Therefore, a direct comparison may not be suitable for assessing model utility.
    $$M/P_{\mathrm{phase}}=\text{ln}f_{u,p}+\text{ln}M_{u}/Pu+\text{ln}K$$ (10)
    The definition of K (milk: lipid partition coefficient) is the same as described for Equations 8 and 9. The regression was carried out for acidic (Equation 11) and basic (Equation 12) compounds separately, as follows:
    $$\text{Acidic Drugs: ln}M/P=-0.405+9.36\text{ln}(M_{u}/Pu)-0.69\text{ln}f_{u,p}-1.54\text{ln}K$$ (11)
    $$\text{Basic Drugs: ln}M/P=0.02477+2.28\text{ln}(M_{u}/Pu)-0.886\text{ln}f_{u,p}-0.505\text{ln}K$$ (12)
  The log-transformed phase distribution model explains a high magnitude of variance in the dataset tested. Therefore, when seen in the context of experimental error and biological variation, the results suggest that the log-transformed phase distribution model takes into account the key determinants of drug distribution into human milk (53). This model is helpful for identifying the drugs which may have potential for partitioning into milk and require further research, in addition to its predictive ability.
  4. Quantitative Structure Activity Relationship (QSAR) Models
    The above mentioned phase distribution models require multiple input parameters. Knowledge of the amount of drug bound to protein and fat, the milk-lipid plasma partition coefficient as well as the fraction unionised in plasma and milk is needed. Although drug $pK_{a}$, plasma and milk pH and plasma protein binding are known for most compounds, the human milk-specific characteristics rely heavily on *in vitro* experimentation.
    QSAR models utilize chemical and molecular properties of drugs to predict M/P. These methods are considered an alternative to the phase distribution models. Agatonovic-Kustrin et al. developed a 26-descriptor nonlinear computational neural network to estimate M/P values, which does not require experimental parameters (55). A Multilayer Perceptron (MLP) architecture has been utilized where the inputs are connected to the hidden layer which is subsequently connected to the output. The output is computed as a sum of non-linear transformations of the linear combinations of inputs. A set of 60 compounds was obtained from the literature, along with the corresponding M/Ps. Agatonovic-Kustrin et al. compared the genetic neural network they built with the log-transformed phase distribution model (Equation 10). Although the log-transformed model predicts M/P with strong correlation to observed M/P, it demonstrates systematic error and method bias, which were overcome by the neural network. They further improved this model by incorporating a set of 123 compounds and encoded each compound with 71 structural descriptors (56). A nine-descriptor nonlinear neural network was selected for the estimation of M/Ps. The key advantage of their subsequent Quantitative Structure Property Relationship (QSPR) model is the prediction of an M/P using the structure of the molecule.
    Zhao et al. described the prediction of M/P using a classification method called Support Vector Machine (SVM) (57). This model was constructed to distinguish the potential risk of drugs to nursing infants. Each drug was represented by a large pool of descriptors, of which five descriptors (logP, Randic index, Maximum partial charge for a C atom, Min E-E repulsion for a C-C bond and Min coulombic interaction for a C-C bond) were found to be most important for the construction of predictive models. The classification accuracy of the training set and the test set for SVM was 90.63% and 90.00% respectively. This indicates that the SVM model acts as a selective filter to evaluate the risk for drugs when experimental M/Ps have not been investigated.

### Limitations and considerations for empirical models

Empirical models can be utilized to evaluate potential risk of drugs to partition into human milk. Empirical modeling approaches encompass unbound drug distribution, membrane diffusion, phase-distribution and the machine-learning based QSAR models. These models have proven instrumental in predicting the M/P, however it is imperative to acknowledge their inherent limitations. Despite their efficacy in capturing complex relationships between variables, the models often rely on assumptions that may not universally hold across diverse datasets or experimental conditions. They rely on the fundamental assumption of passive diffusion of drugs from plasma to human milk. However, a study conducted by Koshimichi et al. found that secretion clearance ($CL_{\sec}$) and re-uptake clearance ($CL_{\mathrm{re}}$) of most unbound drugs between plasma and human milk to be essentially equivalent (58). This suggests that drug transport through mammary epithelia occurs primarily through passive diffusion, as is accepted generally (59).

Therefore, this finding validates the assumptions held by empirical models. Although the Koshimichi et al. study also found certain drugs with dissimilar $CL_{\sec}$ and $CL_{\mathrm{re}}$ values, indicating that passive diffusion is not sufficient to explain drug transport between plasma and milk (58). Therefore, transporter mediated mechanisms need to be incorporated for those drugs in particular.

Moreover, interpretation of M/P obtained by these models cannot be viewed by themselves, but rather as a tool to set up further validation studies. Empirical models enable the prediction of M/P to an approximate value, but are still crucial to facilitate rational decision making regarding breastfeeding for most drugs (53). These M/P prediction tools can make it easier to simulate drug levels in human milk after drug administration to mothers by integration into a PBPK model. The precision of the models mentioned above is sufficient to be used as input for PBPK. Then, if clinical lactation data for a drug is absent, it may be possible to estimate the IDD and RID of a medication based on output of these physiological models and utilize the output to make informed decisions on infant risk assessment (60).

## Pharmacokinetic modeling

Pharmacokinetic model based approaches can provide information on lactational exposure of drugs when direct clinical data are not available. Since the analysis can be performed entirely *in-silico*, PK models can be used to estimate the RID. Model-based approaches have also been utilized to estimate harm and set exposure limits for environmental contaminants in human milk (61–63)

### Physiologically based pharmacokinetic models

**Figure 3:**
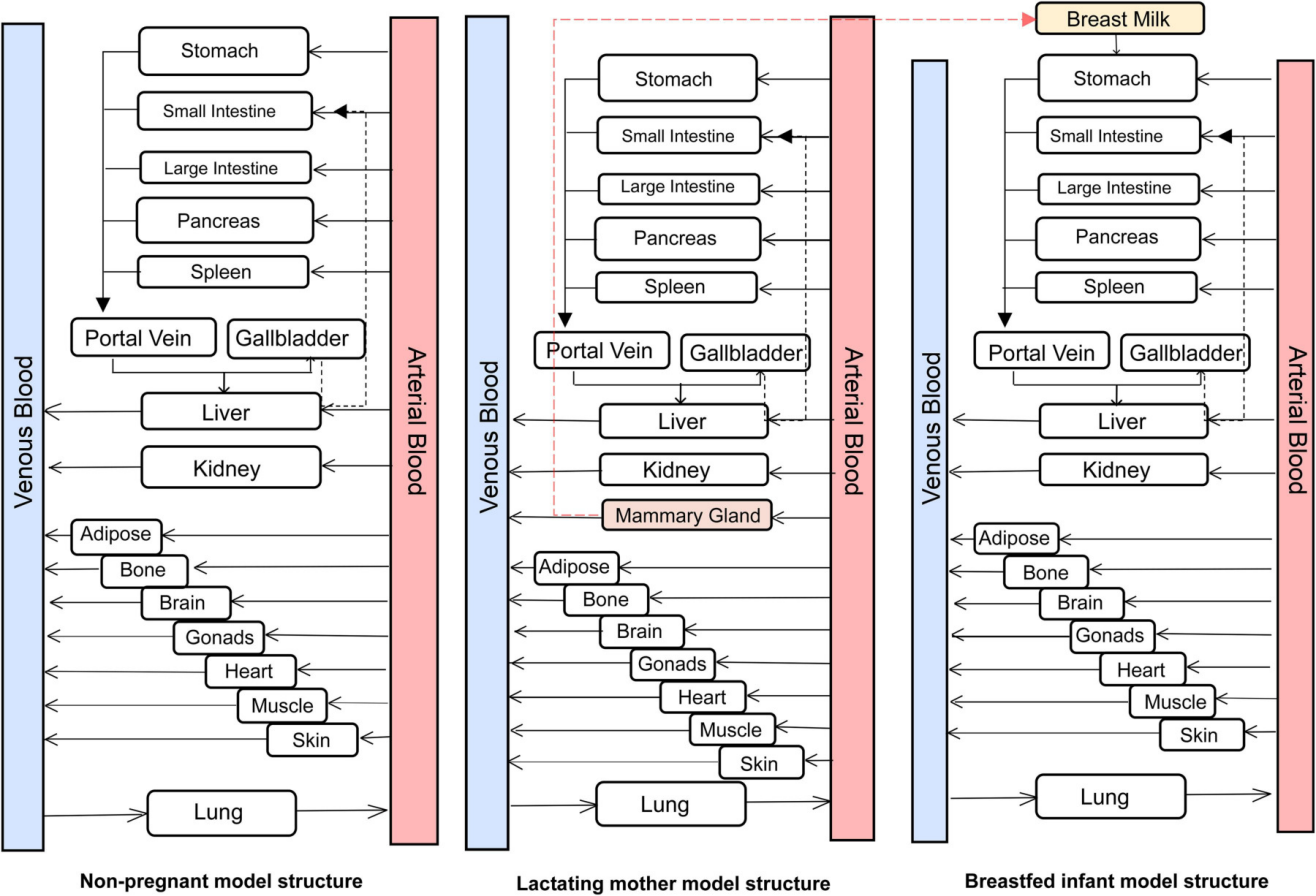
Schematic representation of physiologically based pharmacokinetic (PBPK) model structures used to describe drug disposition across non-pregnant, lactating mother, and breastfed infant systems. The model incorporates physiological compartments linked by arterial and venous blood flow, with explicit representation of the mammary gland to enable simulation of drug transfer into breast milk and subsequent infant exposure.

PBPK models are multi compartmental mechanistic models in which each compartment corresponds to one or more organs and is interconnected by the blood flow. It integrates important physiological parameters (eg. blood flow, abundance of enzymes and transporters, cardiac output, glomerular filtration rate) and drug-related parameters (blood-to-plasma ratio, plasma protein binding, permeability, solubility, *in vitro* metabolism or transport) (64). In addition to physiological and drug-specific inputs, PBPK models require information on study design parameters, including dosing regimen, route of administration, and the timing and nature of biological sampling, in order to generate simulations that are directly comparable to clinical observations. An adult PBPK model can be modified to predict the pharmacokinetics of medicines during pregnancy and lactation or in the developing infant and child by inclusion of the physiology of pregnancy, lactation and childhood growth (65). This involves developing a PBPK model in healthy volunteers which can be modified to represent the lactating women. This model is then coupled to an infant PBPK model. Figure 3 shows the structure of a lactation PBPK model in which a maternal PBPK model is coupled to a neonatal PBPK model to predict milk transfer and neonatal systemic exposure via lactation. The simulated concentration of drug in the milk is used as the dose that the infant receives via lactation to predict exposure in the infant. PBPK models are especially advantageous to incorporate dynamic mechanistic characterization of milk-to-plasma transfer of a drug. Moreover, in order to predict drug concentrations in infant plasma, different infant maturation processes can be integrated to improve accuracy of predictions. PBPK models can be built in commercial : Simcyp Simulator (Certara UK)—and open source platforms : PK-Sim (Open Systems Pharmacology, Germany), mrgsolve [Baron K, Metrum Research Group (66)].

Three approaches have been implemented in the prediction of infant exposure using PBPK based on the method of drug uptake into breast milk from plasma. The first approach considers the direct transfer of the medication from blood into the breast milk similar to PBPK modeling developed to evaluate neonatal exposure to organic pollutants (67). The second approach considers uptake of the medication into breast milk via mammary adipose tissue (68). The third approach combines the above two methodologies (62). All three approaches have been used for chemical substances. Majority of the current PBPK models consider passive diffusion of drugs into the milk with the exception of the lactation PBPK model for five Breast Cancer Resistance Protein (BCRP) substrates (Acyclovir, Bupropion, Cimetidine, Ciprofloxacin, Nitrofurantoin) which incorporates BCRP mediated transport kinetics (69).

PBPK modeling has been used to quantitatively predict RID of potentially toxic chemicals that could result from inadvertent maternal exposure via occupation or environment. Loccisano et al. developed a PBPK model to determine exposure of Perfluorooctanoic Acid (PFOA) and Perfluorooctane Sulfonic Acid (PFOS) in fetus and in infant through milk by extrapolating a previously developed and validated model in rats. Additional information on drug elimination kinetics in fetus and infant can be added as it becomes available for better prediction of drug exposure in neonates (61). Byczkowski and Fisher extrapolated a PBPK model for TCE in rats to predict the extent of an accidental acute exposure of lactating women to TCE (70). Dosing predictions from the PBPK model were utilized to assess neonatal risk of exposure to TCE by input into an equation obtained from the U.S. EPA (71). Merrill et al. developed a PBPK model using data from previous models in male and lactating rats to predict perchlorate and iodide kinetics and subsequent perchlorate induced inhibition of iodide uptake in lactating mothers. The extrapolated human model was validated using radioiodide data from lactating women and nursing infants assuming that it will reasonably estimate human perchlorate kinetics since it was able to describe both anions in rat (72, 73). Published toxicological lactation models have been summarized in Table 6.

**Table 6:** PBPK models for environmental chemicals.

Name of compound	Species	Software used	Model predicted parameters	Potential limitations	Refs.
Methylmercury	Human	Not mentioned	77.2% excretion via hair and 14.9% via human milk	Partition coefficients and kinetic parameters for the fetus and infant were assumed to be similar to pregnant women.	(68)
				Methylmercury calculated as difference between total mercury and inorganic mercury	
Tetrachloro-ethylene (TCE)	Lactating rats and Human	SIMUSOL-V (Fortran-based simulation language)	Model described distribution of inhaled TCE in lactating rat blood and milk as well as nursed pup’s gastrointestinal tract (GI), blood and tissue.	Limited human data for validation.	(70)
Perfluorooctanoic acid (PFOA) and Perfluorooctane sulfonate (PFOS)	Human	acs1X (version 3.0, AEgis Technologies Group, Huntsville, AL)	Simulated PFOA and PFOS concentrations in fetal, infant, maternal plasma and milk.	Limited human data for validation.	(74)
				Infant treated as a single lumped compartment	
Perchlorate and Iodide	Human	Advanced Continuous Simulation Language (ACSL™)	Serum and Urinary $ClO_{4}^{-}$ from drinking water- 0.007 to 12 mg ${\text{ClO}}_{4}^{-}/\text{kg/day}$	Model validation only performed for radio iodide in lactating women	(73)

PBPK modeling for drugs in human milk has been reported only for a few drugs that have some human milk secretion data available to validate the findings. The PBPK models for alprazolam, caffeine, tramadol, digoxin, venlafaxine, fluoxetine and paroxetine only consist of a maternal PBPK model and thus can only predict transfer to milk (75, 76). Also, the utility of these models is restricted to basic compounds with physicochemical properties similar to the compounds for which these models have been developed. The lactation PBPK models for codeine, escitalopram, isoniazid, ethambutol and rifampicin use different approaches to couple the maternal and neonatal models to predict drug exposure in the infant via milk intake. Delaney et al. analyzed the escitalopram concentrations in the milk using a PopPK approach. The IDD was calculated based on the escitalopram $C_{\mathrm{milk}}$, $V_{\mathrm{milk}}$ and feeding frequency (Equation 1). The estimated IDD was then used as the dose input for the neonatal PBPK model (77). The model also evaluated the differences in concentration between hindmilk and foremilk. Escitalopram concentrations were higher in hindmilk but the difference in concentration was approximately 10% suggesting no substantial impact of sampling phases on exposure. Willmann et al. used a similar approach but the medication was administered to the neonatal PBPK model as multiple doses given every 3-h (78). In the PBPK model for ethambutol and rifampicin, the breast compartment was included as a reservoir (Figure 1) and excretion into the reservoir was calculated by multiplying $V_{\mathrm{milk}}$ with $C_{\mathrm{milk}}$ (Equation 1). The milk concentration was estimated using the M/P (79). The PBPK models for codeine and isoniazid also evaluated the impact of genotype on maternal and neonatal exposure (78, 80). The polymorphic expression of isoniazid metabolizing enzyme N-acetyltransferas was not likely to cause any clinically significant adverse effects in nursed infants. However, neonates of mothers with ultra rapid CYP2D6 phenotype may have greater risk of opioid poisoning. Currently published human lactation PBPK models are summarized in Table 7.

**Table 7:** Physiologically based pharmacokinetic models for drugs in human lactation.

Name of drug	M/P determination method	Software used	Model predicted parameters	Potential limitations	Refs.
Tramadol (T), Venlafaxine (V), Fluoxetine (F), Paroxetine (P)	Log-transformed phase distribution model (Section 3c)	Simcyp Simulator v20	Maternal $C_{\mathrm{avg}}$, IDD, RID, M/P ratios: T-1.93, V- 2.23, F- 0.73 , P- 0.61 Within 2-fold range of observed values.	Only for basic drugs	(76)
				Postpartum model unchanged from non-pregnant maternal physiology	
				Considers passive diffusion only	
				Model does not account for dynamic changes in milk composition and volume.	
Actaminophen (AC), Alprazolam (AL), Caffeine(C), Digoxin (D)	Fleishaker model (Section 3a)	Simcyp Simulator v20	Maternal $C_{\mathrm{avg}}$, IDD, RID, M/P ratios: AC-0.83, AL- 0.45, C- 0.69 , D- 0.76 Within 1.26-fold range of observed values.	Considers passive diffusion only	(75)
				Model does not account for dynamic changes in milk composition and volume.	
Acyclovir (A), Bupropion (B), Cimetidine (CM), Ciprofloxacin (CP), Nitrofurantoin (N)	Bottom up and top-down modeling approaches using literature reported data for optimization	Simcyp Simulator V21	Maternal $C_{\mathrm{avg}}$, M/P ratios: A-2.48, B- 3.70, CM- 3.55 , CP- 1.21, N- 5.78 Within 1.5-fold range of observed values.	Degree of BCRP induction not known- Results should be interpreted with caution	(69)
Efavirenz	System and drug related parameters for infant obtained from literature or scaled from adults	SimBiology (v 5.1, MATLAB 2014b)	Maternal $C_{\max}$, ${\text{AUC}}_{0-24}$, $C_{\min}$ and Infant $C_{\mathrm{avg}}$ , Maximum Infant Dose and Infant Exposure Index: 5.9% at 400 mg, 8.7% at 600 mg Within 2-fold range of observed values.	Considers passive diffusion only	(81)
				Ontogeny of drug metabolizing enzymes in infants not known	
Escitalopram	Not determined	PK-Sim Ⓡ v6.3	Maternal $C_{\mathrm{avg}}$, IDD, RID, Infant Exposure Index: 1.7% Observed mean values within 95% confidence interval of predicted.	Ontogeny of drug metabolizing enzymes in infants not known	(77)
Isoniazid	Not determined	deSolve in R version 3.4.1.	Maternal $C_{\max}$, ${\text{AUC}}_{0-24}$, $C_{\min}$ and Infant $C_{\mathrm{avg}}$ , RID and Oral exposure to infant after 900 mg maternal dose: Fast metabolizing mother- 1.75 mg/day, Slow metabolizing mother- 4.46 mg/day	Considers passive diffusion only	(80)
Ethambutol (E) and rifampicin (R)	Literature values incorporated in model	MATLAB 8.0	Infant $C_{\mathrm{avg}}$ and RID: E- 0.08 mg/kg body weight/day, R- 0.4 mg/kg body weight/day	Infant clearance scaled from adults	(79)
Morphine, Codeine	Literature values incorporated in model	PK-SimⓇ v4.0	Infant $C_{\mathrm{avg}}$ and RID: Codeine- 0.38 mg/kg, Morphine- 0.17 mg/kg	Conversion pathway of codeine to morphine has only been considered	(78)

### Population pharmacokinetic models

Traditional PK studies in lactating women present many practical and ethical constraints, making it challenging to examine the inter individual variations in drug secretion into human milk and subsequent infant exposure. However, population pharmacokinetic (PopPK) studies are based on sparse sampling from a reasonably large population and are ideal for simulation analyses of variability in drug exposure levels in lactating women and their infants (21, 82, 83). The PopPK approach provides estimates of infant exposure to drugs in human milk. This approach assumes that the human body is divided into linked compartments which comprise of kinetically homogeneous body parts. In determining infant exposure, milk is considered as the peripheral compartment connected to the maternal plasma in the central compartment with direct exchange between plasma and human milk. Many lactation PopPK models detailed in Table 8 utilize a previously published non-pregnant or pregnant PopPK model and link maternal human milk concentrations using an M/P.

**Table 8:** Population pharmacokinetic models for lactation.

Name of drug	Structural model	Covariates included	Model predicted parameters	Potential limitations	Refs.
Bedaquiline	3 Compartment (CMT) for parent and 1 CMT for metabolite	PK study, Dose	Model estimates: M/P- 13.6 (Parent), 4.84(M2 metabolite), Maternal $C_{\mathrm{avg}}$-0.4 mg/L, RID- 0.816 mg/kg/day.	PK in postpartum patients assumed to be the same as non-pregnant patients.	(86)
				Unbound concentrations not measured.	
Dolutegravir	2 CMT (Maternal) and 1 CMT (Infant)	No significant covariate identified	Model estimates: M/P (maternal plasma)- 0.033, Median infant $IC_{90}=0.064$ mg/L	Dolutegravir dose at delivery not known.	(87)
				Gestational age at delivery not recorded.	
Fluoxetine (FX), Norfluoxetine (NFX)	1 CMT with first order absorption, FX-NFX conversion coefficient	None included	Model estimate: RID- 0.028 for FX, 0.029 for NFX	Small sample size (n=24).	(83)
				CYP2D6 polymorphism not considered.	
Azithromycin (AZI)	3 CMT with mixed zero-and first-order absorption	No significant covariate identified	Estimated total absolute IDD = 4.5 mg/kg/day, Median RID = 15.7%	Evident time-dependant change in M/P: not reliable for estimation	(88)
				Prophylactic AZI in milk may be of benefit, but potential adverse effect to infants	
Tramadol and its O-desmethyl Tramadol (ODT)	5 CMT (Dose/Gut CMT included) with first-order absorption and first-order elimination	Creamatocrit	Mean Tramadol RID: 2.16% Extensive Metabolizers, 2.6% Poor Metabolizers	Only sparse data utilized: one sample per subject	(89)
				Most parameters fixed based on literature values	
Allopregnan-olone (Measured), Brexanolone (Administered)	2 CMT with linear elimination and distribution	Maternal body weight	Estimated median RID: 0.69%	Lipophilic drug- lipid content of milk not considered.	(21)
				Milk production prior to brexanolone treatment not assessed.	
Escitalopram (SCIT)	1 CMT with first-order absorption and elimination	Milk fat content, Maternal CYP2C19 Phenotype	Estimated RID: 3.3%	Limited sample size (n=33).	(90)
				Limited adherence- potentially underestimated RID.	
Piperaquine	Sigmoid $E_{\max}$ model	No significant covariate identified	Median Estimates: Absolute daily dose = 0.41 μg/kg, RID = 0.004%	human milk samples not normalized to the time of dosing.	(91)
				Few maternal plasma samples- impacting M/P estimation.	
Praziquantel (PZQ)	3 CMT with first order absorption (lag function applied) and linear elimination	No significant covariate identified	Estimated Median IDD: 0.037 mg/kg	PZQ allowed to redistribute to maternal plasma without terminal elimination.	(92)
				Secretion of PZQ in human milk assumed to be minimal.	

Software programs such as NONMEM (ICON LLC), Monolix (Simulations Plus, Lancaster CA), Phoenix WinNonlin(Certara UK) and the R package Pmetrics (84) can be used for population pharmacokinetic modeling purposes. These parametric and non-parametric software programs can simultaneously provide information on parameters estimates, inter individual variability and residual error. We have summarized the currently published human lactation PopPK models in Table 8.

The first step in PopPK model development is identifying the structural model which best describes the pharmacokinetics of the drug and the observed inter individual and residual variability followed by covariate analysis which allows for the detection of clinically, biologically and demographic plausible factors associated with such variability (85). Typically tested covariates include maternal age, body weight, albumin and alpha-1-acid glycoprotein (AAG) concentration, CYP polymorphisms, milk composition (fat, protein and carbohydrate) and feeding occasion (hindmilk vs. foremilk). However, based on the studies included in Table 8, most lactation PopPK models do not find these covariate relationships significant for inclusion into the final model.

Unlike PBPK modeling, population pharmacokinetic modeling requires clinical data from at least a milk-only study to predict lactational exposure (93). Population modeling has the advantage of handling variability in sample collection times allowing between deviations from study protocols (94). This is advantageous especially in the nursing phase where nursing mothers are taking care of the infant while participating in clinical studies.

Data from two conventional pharmacokinetic studies were used to build a robust model to estimate the pharmacokinetic parameters of fluoxetine and norfluoxetine in milk. However, a scaling factor was used to obtain norfluoxetine concentrations and the polymorphism and ontogeny of CYP2D6 was not accounted for in the model (83). Similarly, data from the DolPHIN-1 study which investigated dolutegravir pharmacokinetics in women with untreated HIV late in pregnancy was used to develop a population pharmacokinetic model to predict infant exposure. Similar plasma exposure was observed during third trimester of pregnancy and postpartum and the human milk transfer of dolutegravir was negligible (87). The population PK model can be used to perform simulations to calculate and obtain an estimate of the range of RID. This involves simulating the maternal concentration profiles in human milk for a large number of mother-infant pairs which allows for the prediction of variability in drug transfer through milk and subsequent infant exposure. Monte Carlo simulations (95) were performed with the population pharmacokinetic model of fluoxetine to obtain an estimate of the spread of RID values in 1,000 simulated infants (for internal validation of the model) (83). Similarly, simulations were performed in 1,000 simulated lactating women to estimate the partitioning of praziquantel in human milk (92). However, these simulations can also be performed to estimate infant exposure across different dosing situations or feeding patterns. For example, model-based simulations were performed for piperaquine treatment given at birth, 1 and 6 weeks post delivery in Melanesian mothers. The relative total infant dose was highest when piperaquine was dosed 6 weeks postpartum, but it was still below the 10% threshold (91).

Lactation PBPK and PopPK models developed over the past several years have been able to reasonably predict infant exposure. When there is absence of data during pregnancy and lactation, such models can definitely help to provide an estimate of infant exposure. However, there are several shortcomings at the current time. There is a lack of data on drug metabolizing enzymes and transporters present in breast tissue, and hence the majority of the models only consider passive diffusion of drugs into the milk, which may be accurate for most drugs as mentioned earlier. Current models estimating infant exposure only consider drug transfer through milk as the route of exposure but do not account for *in utero* exposure to drugs. The inherent nature of these models cannot account for inherent toxicity of medications or non-dose related adverse effects. Despite these limitations, predictive modeling approaches are continuously improving. The integration of empirical and pharmacokinetic predictive modeling tools provide an essential framework to understand drug secretion into human milk, given the dearth of knowledge in this area.

## Conclusion

The current literature review highlights several overarching conclusions regarding the study of drug transfer into human milk. First, commonly used exposure metrics such as milk-to-plasma ratio and relative infant dose are informative but inherently context dependent, and should not be interpreted in isolation when assessing infant risk. Second, non-clinical models provide valuable mechanistic insight and support early risk prioritization, but their quantitative extrapolation to humans is limited by substantial species- and system-specific differences. Third, while clinical lactation studies remain the most reliable source of evidence, their design must balance scientific rigor with feasibility, emphasizing pragmatic, longitudinal, and opportunistic sampling strategies. Finally, predictive modeling approaches—including empirical equations, population pharmacokinetic models, and physiologically based pharmacokinetic models—offer the greatest potential to bridge persistent evidence gaps by integrating experimental data with physiological context. A critical comparison of these three modeling paradigms reveals distinct trade-offs. Empirical models are the most accessible and require only physicochemical drug properties as inputs, but assume passive diffusion and offer limited mechanistic insight; their predictive performance is compound-dependent. Population PK models excel at characterizing inter-individual variability from sparse clinical data but depend on the availability of at least some clinical observations and offer limited extrapolation beyond the studied population. PBPK models are the most mechanistically rich and support prospective simulation in the absence of clinical data, but require the greatest number of input parameters and are sensitive to assumptions about mammary physiology and transporter expression. Importantly, the practical selection of modeling framework should be guided by the available data, the research question, and the stage of drug development. Recent work integrating empirical predictions as informative priors within PBPK or PopPK frameworks represents a promising hybrid strategy that may improve predictive accuracy while retaining mechanistic interpretability. Continued refinement of these models will require improved understanding of time-varying milk composition, mammary transport processes, and infant developmental pharmacology. Collectively, the integration of experimental and model-based approaches represents the most robust and ethically feasible path forward for informing medication use during lactation and improving evidence-based guidance for breastfeeding individuals.
